# Mind the gap: analysis of two pilot projects of a home telehealth service for persons with complex conditions in a Swedish hospital

**DOI:** 10.1186/s12913-023-09409-4

**Published:** 2023-05-09

**Authors:** Carla Sacchi, Karolina Andersson, Marta Roczniewska, Jamie Linnéa Luckhaus, Moa Malmqvist, Lars Peter Rodmalm, Karin Lodin, Rebecca Mosson, Petra Danapfel, Carolina Wannheden, Pamela Mazzocato

**Affiliations:** 1 Department of Business Studies, Uppsala University, Uppsala, Sweden; 2 Research, Development, Education, and Innovation Unit, Södertälje Hospital, Södertälje, Sweden; 3 Medical Management Centre, Department of Learning, Informatics, Management and Ethics, Karolinska Institutet, Tomtebodavägen 18A, Stockholm, 171 77 Sweden; 4 Institute of Psychology, SWPS University of Social Sciences and Humanities, Warsaw, Poland; 5 Department of Women’s and Children’s Health, Uppsala Universitu, Uppsala, Sweden; 6 Department of Medicine, Karolinska Institutet, Stockholm, Sweden; 7 Change Management and Processes Unit, Region Östergötland, Linköping, Sweden

**Keywords:** Complexity, Quality improvement, Innovation, Telehealth, Heart failure, COVID-19

## Abstract

**Background:**

Developing and implementing home telehealth (HTH) services for patients with chronic conditions is a challenge. HTH services provide continuous and integrated care to patients, but very often pilot projects face non-adoption and abandonment issues. Change processes in healthcare are often complex and require learning to adapt to non-linear and unpredictable events. Complexity science can thus provide a complementary view to the predominant Quality Improvement (QI) approach in healthcare. In this study of two pilot projects in a Swedish hospital, we explore how a theory-driven approach can be used (a) to support the development of a self-monitoring HTH service in hospital care and (b) to evaluate staff and patients’ experiences from early adoption.

**Methods:**

To plan and evaluate the service for the recipients (i.e., patients and healthcare providers), we used the Plan-Do-Study-Act (PDSA) tool in combination with two complexity-informed frameworks: the Non-adoption, Abandonment, Scale-up, Spread and Sustainability (NASSS) framework, and the joint Complexity Assessment Tool (CAT). The theory-informed development process led to two pilot projects of an HTH service for patients with heart failure and COVID-19. We collected data from multiple sources (project documents, a survey on readiness for change among staff, and semi-structured interviews with patients and staff) and analyzed the data using descriptive statistics and qualitative content analysis with a deductive approach.

**Results:**

Patients and staff perceived the services as valuable as they enabled rapid feedback, and improved communication and collaboration between patients and healthcare providers. Yet, despite the extensive development efforts, there was a perceived gap between how individuals valued the service and the capacity of adopters, the organization, and the wider system to effectively integrate these services into routine care.

**Conclusions:**

The combined use of PDSA, NASSS, and CAT can support the development and evaluation of HTH services that are perceived as valuable by individual patients and staff. For successful adoption, the value for individuals must be supported by organizational efforts to learn how to integrate new routines and tasks into clinical practice and daily life, and how to coordinate multiple providers within and outside the hospital walls.

**Supplementary Information:**

The online version contains supplementary material available at 10.1186/s12913-023-09409-4.

## Background

“Ethel is an older woman with heart failure and chronic obstructive pulmonary disease. She lives alone in an apartment and receives home care. The last time Ethel went to the hospital, she had shortness of breath and clearly rumbled. She felt scared. The first time she had similar problems, she felt ignored at the primary care center, but a nurse arranged for her to be sent to the hospital. So, Ethel now feels uncertain: when are the problems ´real´ and when should she wait? From her last time in the hospital, she remembers that she felt well taken care of. However, it did not feel safe to be discharged because she still felt too ill to go home. No one had really told her about her illness or what symptoms she should pay attention to. When she came home from the hospital, she had wanted someone to contact her to find out how she was feeling or if she needed anything. Instead, she came home to an empty and quiet apartment where she had to take care of most things by herself.” This narrative comes from an interview conducted during a feasibility study at Södertälje Hospital in Sweden in 2019. The story of Ethel is not unique for patients with chronic conditions [1]. Such conditions can be considered complex both from clinical and managerial perspectives, since often patients present multiple chronic conditions and thus it is challenging to classify them into clear disease groups, which in turn creates inconsistencies and a lack of established standards to manage their care [1, 2]. The amount of stress and uncertainty experienced by patients with chronic conditions when going through fragmented and incoherent care pathways is typically underestimated [3]. Patients may experience low levels of control in a life filled with pills, injections, and appointments with a variety of healthcare staff at different levels of care. Worsening of disease symptoms and associated feelings of worry and anxiety can trigger unplanned readmissions, and if these factors are detected at an early stage, readmissions could be prevented [4].

Home telehealth (HTH) services can enable hospitals to expand their capacity to provide care beyond their walls and enable providers and patients with chronic conditions to combine resources and improve outcomes and patient experience throughout the patient’s life-long disease journey [5]. HTH services, often based on the use of wearable self-tracking devices, generate independent and automated measurements of patients’ health status that allow providers to understand when and how to get involved in a patient’s care. Nevertheless, there are still questions about how they can be effectively implemented in hospitals. The value of HTH is dependent on the ability to integrate it into current healthcare systems and routine care practices [6]. Research has shown that implementation of such services is challenging, which is reflected in the frequent non-adoption and abandonment of HTH services [7], and in most of the projects not being implemented after the pilot stage [6, 8].

Change processes in healthcare are often chaotic, characterized by unexpected events, discontinuous activities, and shifting goals [9]. Quality improvement (QI) is one of the predominant approaches to change in healthcare. QI focuses on understanding, reviewing, and revising processes using data. One of the most common tools in QI is the Plan-do-study-act (PDSA) improvement cycle [10]. Nevertheless, the effectiveness of different tools and methods, such as PDSA, are continuously debated and their application in complex healthcare contexts can be difficult [10, 11]. Complexity science can offer a complementary view to QI regarding the dynamics of developing and implementing HTH services for patients with complex conditions.

Healthcare organizations have been characterized as complex organizations that are set in complex, changing systems [9]. In such systems, successful implementation requires collaboration and alignment of different players in a networked way, and continual adaptation of an intervention to the context and the priorities of the organization and its users [12]. Organizational change is seen as a non-linear and unpredictable process, and approaches that make space for co-evolution, self-organization, and emergence are better suited to respond to the complex dynamics of change [9, 13]. Thus, there is a need to better understand how implementation processes can promote a dynamic interaction among contextual factors such as individuals, processes, and organizations, to support the successful adoption of HTH services into clinical practice.

The framework for Non-adoption, Abandonment, Scale-up, Spread and Sustainability (NASSS) addresses the complexity of technology adoption in healthcare and helps to explain failure in sustainable implementation [14]. The framework emphasizes the complex interactions between seven domains (and related sub-domains) that influence the adoption of e-Health services in healthcare [15]: D1) Medical condition (nature of the illness, comorbidities, socio-cultural factors); D2) Technology (material properties, knowledge to use it, knowledge generated by it, supply model, intellectual property rights); D3) Value proposition (value for the supplier and for the patient); D4) Adopters (staff, patients, carers); D5) Organizational readiness (capacity to innovate, readiness for this technology, nature of adoption, extent of change, work needed); D6) Wider system (political, regulatory and sociocultural context, interorganizational networking); and D7) Embedding (adaptation over time, organizational resilience). Based on the dynamic interactions among these seven domains, a distinction can be made among simple, complicated, and complex systems [14]. Simple systems have a few components that interact in predictable ways. Complicated systems have multiple components that also interact predictably. Complex systems have multiple, intricately related components that are characterized by constantly changing, unpredictable, and non-linear interconnections.

The NASSS framework was later integrated with a Complexity Assessment Tool (CAT), which includes three dimensions of complexity (structural, socio-political, and emergent) and aims at anticipating and actively managing these dimensions [16]. A suite of NASSS-CAT tools was developed to support the implementation of technology in the different phases of project management [15]. The NASSS-CAT tools contain a list of suggested actions and questions to guide project planning and to monitor complexity of technology implementation over time, in each of the NASSS domains. By applying such tools, project managers can estimate qualitative and semi-quantitative results to assess the complexity levels of the different domains of their project, either before, during or after they are carried out.

The NASSS framework has been used to explain the success and failure of several technology-supported health intervention programmes [17], but only one case [18] that we are aware of used NASSS-CAT prospectively to develop technology-supported services. Thus, the value of the NASSS framework in studies of hospital-based HTH services and in prospective development and implementation processes in a hospital setting remains to be studied. Yet, using this framework in a prospective manner is relevant because it can complement the more established QI approach by embracing the inherent complexity dimension of such processes.

### Rationale and aim

Despite the promises of HTH there is still limited knowledge on how hospitals and other healthcare providers can successfully overcome the complexity described above and adopt HTH services in routine clinical practice and in patients’ everyday life. The present study applies a theory-driven approach (i.e., the combined use of QI and NASSS) to analyze the conditions that may support the adoption of HTH services in a hospital setting. The aim of this study is to explore how a theory-driven approach can be used (a) to support the development of a self-monitoring HTH service in hospital care, and (b) to evaluate staff and patients’ experiences from early adoption.

## Methods

### Study design

This exploratory single-case study used a mixed-methods approach [19]. The intervention studied was a QI initiative intended to develop an HTH service for distance monitoring of patients with complex conditions. Originally the targeted patient group was older adults with chronic conditions, and was later extended to patients with COVID-19 infection (see more information below). We used a theory-driven approach to guide the development and evaluation process. The starting point was pre-existing HTH technologies and thus the development process focused mainly on processes (e.g., resources, activities, competences, etc.) that effectively support the adoption of HTH services.

### Context of the intervention

Sweden offers universal health coverage to all residents, and the care system is decentralized, which means that it is managed by each region independently [20]. Specialist and acute care are provided by hospitals, laboratory analyses and primary care are provided by primary care centers, while home care for the older adults and not self-sufficient people is organized by the municipality. Such a division often challenges the integration and continuity of care, especially for patients with chronic or complex conditions [21]. The government identified eHealth as a potential solution to these problems in its “Vision eHealth 2025” [22], where it was suggested that digitalization could re-center care on the individual making it more equal, efficient, accessible, and safe [23]. In addition, efforts have been exerted to plan a system transformation towards coordinated and person-centered care [21].

The present intervention was developed at Södertälje Hospital (SH), a medium-large hospital in the Stockholm Region, in Sweden. SH had 158 available beds in 2019, excluding the intensive care unit, with an average occupancy rate of 100.6 percent, indicating overcrowding [24]. A feasibility study conducted at SH in 2018 found that 251 inpatients had four or more visits per year. This patient group corresponded to 4.6% of all inpatients in medicine and geriatrics and were responsible for 23% of all inpatient visits at the hospital. From interviews with patients with heart failure (HF) and chronic obstructive pulmonary disease (COPD) and their family members, factors that influenced care consumption were identified as: patients living alone, patients experiencing they were discharged too early from the hospital, and the lack of important information on their health and condition. Thus, the need to provide better care before and after discharge was identified and HTH deemed as a possible intervention to avoid unnecessary readmissions and provide better care.

In 2019, the hospital’s management decided to launch a QI initiative to pilot a HTH service for patients with chronic conditions. The goal was to develop and implement a service for treating chronic patients at home before the disease would worsen and the patients require hospital admission. When the COVID-19 pandemic hit Sweden in March 2020 the initiative turned, temporarily, into an effort to use HTH to avoid unnecessary re-admission for patients who had been admitted with a COVID-19 infection.

### The intervention

The intervention took place in two separate pilot projects that resulted in testing technology and processes for HTH for three patients (whereof one female; between 44–60 years of age) with COVID-19 between June and October 2020, and eight patients (whereof one female; between 66–92 years of age) with heart failure (HF) between December 2020 and March 2021. Two additional patients (two female, 50 and 60 years old) were initially included in the COVID-19 pilot project, but dropped out before starting the utilization of the self-monitoring service. One dropped out because of a worsening of the medical condition, and the other decided to withdraw from the project after trying the devices for a couple of days during the hospital stay. The inclusion and exclusion criteria are listed in Table 1.

**Table 1:** Inclusion and exclusion criteria for patient recruitment

	**Inclusion criteria**	**Exclusion criteria**
**For both pilot projects**	- Patients who are judged to be able to participate in distance monitoring - Patients who are judged to be able to benefit from distance monitoring (i.e., patients who have frequent contacts with healthcare) - Patients whose participation in distance monitoring is not judged as potentially harmful for their health - Fluency in Swedish (written and spoken) - Own accommodation - 18 years of age or older	- Cognitive impairment - Impairment due to mental illness - Obstructive hearing or visual impairment affecting the ability to participate in the distance monitoring
**For COVID-19 patients**	- Hospitalized due to COVID-19 infection - Ready to be discharged - Regular values for vital parameters (temperature: 36,1–37,9 °C, respiratory rate: 12–20 breaths/min, oxygenation: 93–100%, systolic blood pressure: 110–190 mmHg, heart rate: 50–100 beats/min) - Up to 5 points on the Clinical Frailty Scale, ranging from 1 (no frailty) to 9 (highest possible frailty) [25]	
**For Heart Failure patients**	- Confirmed heart failure - Symptomatic heart failure regardless of cause (NYHA II-IV) - Out-patients or discharged after being admitted to the hospital	

The technology provider Cuviva® [26] was selected under the provision of the Public Procurement Act (SFS 2016: 1145). Cuviva® provided an online platform called “Remote Patient Monitoring”, used for remote contact between care providers and patients, and to register and store patient data.

The responsible units for the HTH were an inpatient ward (Infections and Internal Medicine unit, IIM) for the COVID-19 pilot project and two outpatient units (Cardiology Unit, CU, and Multimorbidity Unit, MMU) for the HF pilot project. Thus, all staff had to manage their usual patient flow in addition to the HTH patients.

Patients were recruited with a slightly different process by these units. COVID-19 patients and HF patients from the CU were recruited after being admitted to the hospital and HTH equipment and training were provided at the hospital. In contrast, patients recruited by the MMU were offered HTH and received training during a planned visit at the hospital, and the HTH equipment was delivered and set-up in their home by the MMU.

Each patient was provided with a mobile patient unit (tablet), a 4G router for communication, and sensor devices for self-measurements of vital parameters: weight (HF pilot project only), oxygen saturation, heart rate, blood pressure, temperature, respiratory rate. In addition, COVID-19 patients received daily video-calls, from a doctor on the first day after discharge, and from a nurse in the following days. In both pilot projects, daily measurements were taken manually by each patient and values were transferred to the platform, so patients could see their own measurement values in their mobile patient unit and be monitored by hospital staff. If the values were anomalous (outside the pre-defined standard limits), the care provider (nurse or doctor) was able to see it when logging into the system – no automatic notification was sent out. The care provider would then contact the patient via text message, phone or video call. Depending on what emerged during this follow-up, the patient was asked to continue with HTH or visit the hospital for further investigation and care.

HF patients recruited via the MMU could also receive a home visit from a mobile team connected to the MMU during office hours, while home visits were not available for patients recruited via the CU. Initially, for the HF pilot project, the Advanced Hospital Care at Home (AHCaH, in Swedish “Avancerad sjukvård i hemmet”) was supposed to provide assistance and home visits during weekends and nights, but the unit dropped out because of lack of capacity to cover this service. Thus, whenever a serious issue occurred the patient had to go to the Emergency Department.

### The quality improvement process

The development and adoption of the HTH service was conceived as a QI intervention, that is a systemlevel effort to improve the quality, safety, and value of healthcare [27]. The intervention followed an iterative approach in line with the PDSA improvement model [28]. A multidisciplinary project group and a steering committee were formed in both pilot projects and met on a weekly or bi-weekly basis.

**Fig. 1:**
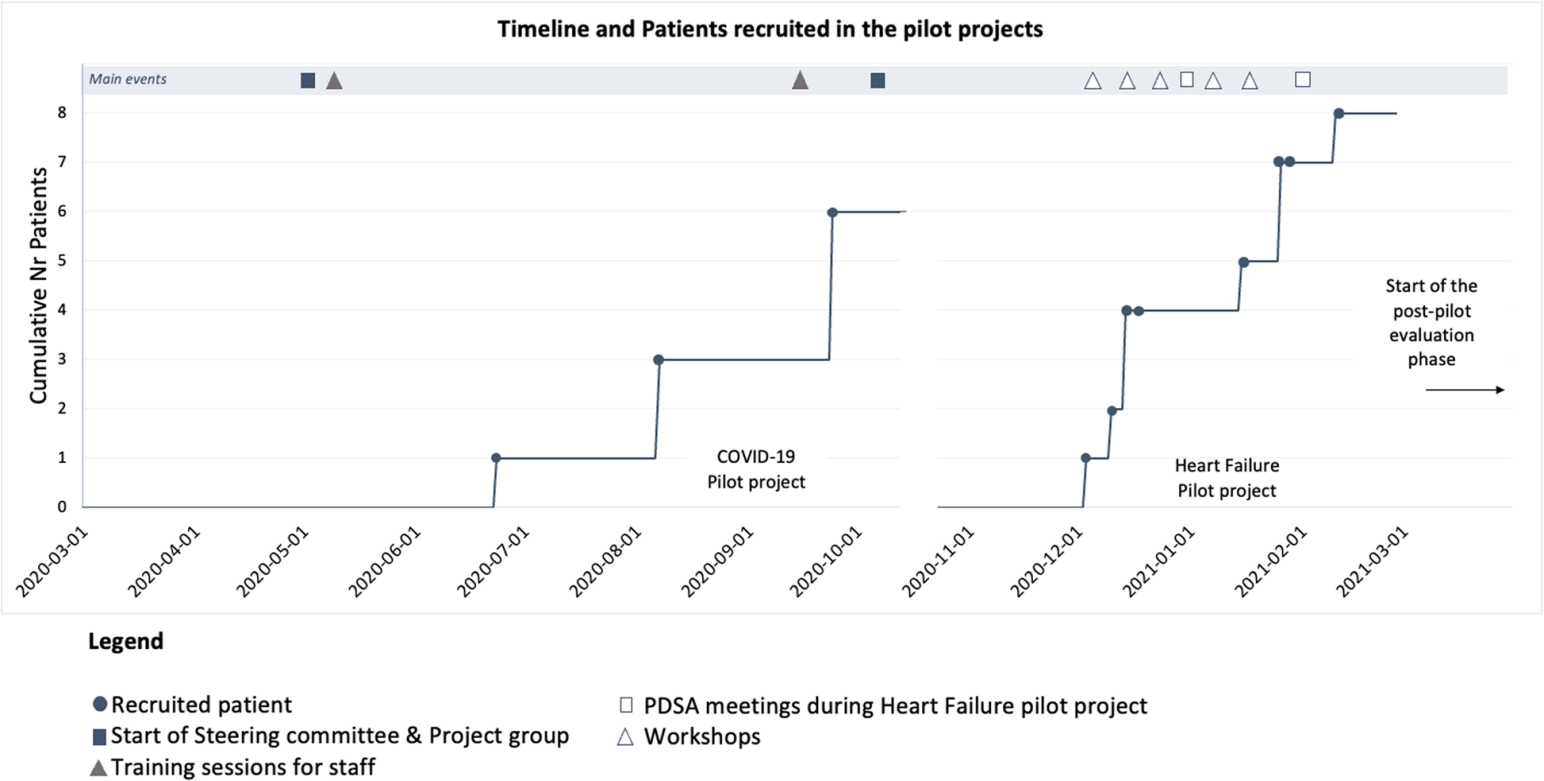
Overview of the quality improvement process timeline

While the first pilot project relied on regular meetings of the project committee, in the second pilot project the four main phases of an improvement cycle (i.e., “plan”, “do”, “study” and “act”) were used to structure the initiative more systematically. Further, more PDSA cycles were then embedded in the “do” phase, in accordance with the iterative nature of this approach. An overview of the whole QI process is presented in Fig. 1.

#### The “planning” phase

The “planning” phase included: (a) workshops to outline the preliminary care process of the HTH (i.e., the main activities and resources involved and to assess the risks associated with the project); (b) risk assessment, and (c) staff training.

**Fig. 2:**
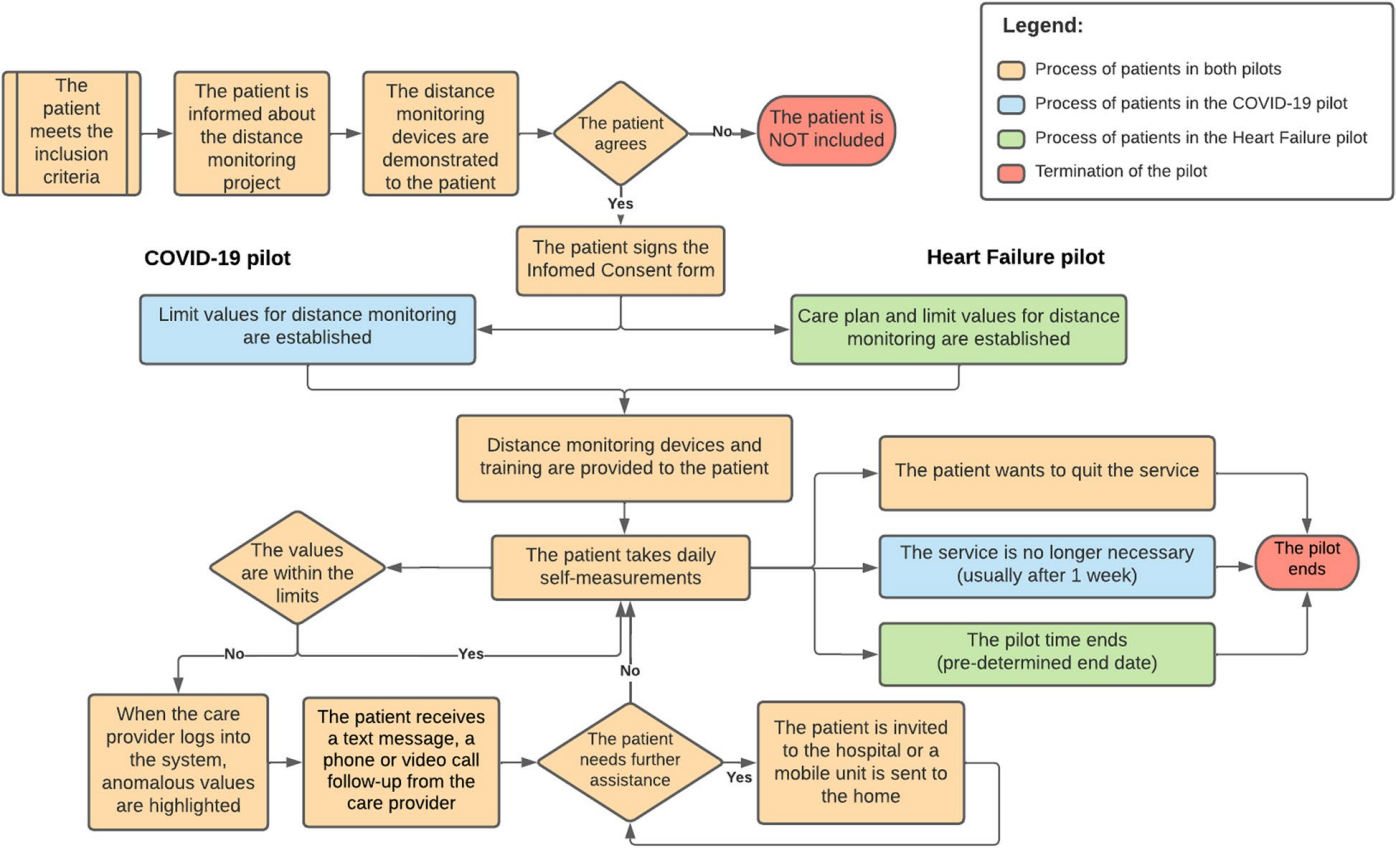
Preliminary care processes

The preliminary care processes as drawn during the planning phase are described in Fig. 2.

The risk assessment identified the lack of standardized routines to track measurement data and other information in the electronic patient medical record as the main risk. Other risk activities related to data protection, information security, continuity, personal data, and confidentiality.

Healthcare professionals (HCP) were provided an on-site 2-h training session by the supplier. For the COVID-19 pilot project, one HCP was trained, who then taught around 20 additional care providers. For the HF pilot project all care providers (around 15) were trained directly by the supplier. Online videos were also made available. After the training, working routines were created.

#### The “do” phase

The “do” phase involved the gradual inclusion of patients. In the HF pilot project, this was carried out through a series of five embedded PDSA cycles, with the aim to systematically reflect on the lessons learned after the inclusion of each new patient and improve the process thereafter. Examples of improvement areas identified through PDSA were the need to support staff in using the technology, beyond the training received, troubles with wireless connection, challenges for patients to answer the daily questions, and the need to use standard care plans to coordinate the work of HCPs.

In the HF pilot project, two of the PDSA cycles also involved two workshops guided by a NASSS-CAT facilitation guide, based on two of the available NASSS-CAT tools (NASSS-CAT LONG and NASSS-CAT PROJECT) to map the complexity domains [15]. The purpose of the workshops was to facilitate a reflection on the use of HTH to inform the further development of the service. The facilitation guide included two parts: (a) an individual survey where participants were asked to agree or disagree with the statements that described the domains of the NASSS framework; (b) a facilitated discussion to explore similarities and differences in the experience of staff. The two workshops were carried out in December 2020 and February 2021 and included five staff members from departments of Information Technology & Medical Technology, Research, Development & Innovation, Geriatrics in the first workshop, and two nurses in the second workshop. During the workshops, some of the same issues identified in the previous PDSA cycles reoccurred. Additionally, the workshops allowed a gathering of multiple stakeholder perspectives and provided a shared understanding of the challenges faced.

#### The “study” phase

The NASSS-CAT PROJECT tool was used in this phase [15]. This tool allowed project managers to collect participants' views on a list of relevant items with a semi-quantitative assessment of the degree of complexity of each item.

Patient’s experiences of HTH were collected through a digital questionnaire, and administered through the HTH platform at the beginning and end of each pilot project. Areas of interest were self-rated health, safety, and accessibility to care. Additionally, six short phone interviews were conducted with the HF patients to better capture their experiences of the service.

The main findings from this phase were used to support this study by providing information on the context and on the QI process.

#### The “act” phase

Following the initial assessment from the first two pilot projects, the hospital joined a regional initiative to develop a regional platform for HTH services. At the moment of writing, the initiative is still ongoing.

### Study of the intervention

#### Data collection

We collected data from documents, notes and recordings taken during the NASSS-CAT workshops, a survey, and individual interviews. Documents from SH, such as project meetings notes, internal evaluations of the two pilot projects, and transcripts from the two NASSS-CAT workshops, were collected to gather data on the improvement process and on the content of the HTH service.

To measure staff readiness to adopt the new technology, we used an eHealth survey (e-Ready) [29]. E-Ready is a self-help tool to guide the implementation of eHealth initiatives, targeting healthcare provider’s readiness and engagement readiness. The tool primarily considers implementation in the local healthcare setting using a stakeholder perspective, and it consists of a readiness assessment survey and a hands-on manual. The survey investigates some key aspects of eHealth readiness divided into the following six sections: (1) Perceived conditions for change at the workplace; (2) Perceived individual conditions for change; (3) Perceived support and engagement among management, (4) Perceived readiness among colleagues; (5) Perceived consequences on status quo; and (6) Perceived workplace attitudes toward change. In addition, seven single items investigate compatibility with current work routines, looking at commitment to change and perceived need for change. We administered the survey to all workers at the departments where the services were planned to be used, for a total of 133 employees in the COVID-19 pilot project and 73 employees in the HF one. The former was administered digitally in June 2020, and the latter on paper in November 2020, prior to starting use the HTH technologies. Responses were collected anonymously, and participation was voluntary. The response rate was 18% for the COVID-19 pilot project and 19% for the HF.

Finally, after completion of the pilot projects, we performed interviews with the following participants: (a) three interviews with COVID-19 patients (summer 2020); (b) four interviews with HF patients (summer 2021); and (c) two interviews with nurses from the HF pilot project (autumn 2021). We used a semi-structured interview guide with open-ended questions based on the NASSS-CAT interview tool [15]. The﻿ interviews were conducted in Swedish by one or two of the authors. Interviews in groups (a) and (c) were conducted online via video-call using the software Zoom®. Interviews in group (b) were conducted in a hybrid form (one interviewer was sitting with the patient, the other was online on the software Zoom). We audio-recorded and transcribed all interviews.

#### Data analysis

We read through collected documents and used them to develop descriptions of how the improvement initiative was organized and how the HTH services were planned and delivered. This data is presented mainly in the methods, but also provided contextual understanding needed to analyze the survey and qualitative data.

We performed qualitative content analysis on the interviews and on the transcripts from the two NASSS-CAT workshops with a deductive approach [30]. We analyzed qualitative data using a codebook developed based on NASSS domains and subdomains [15]. After selecting the meaning units, we condensed them in Swedish to be as close to the original text as possible. We then sorted the condensed meaning units around the NASSS domains and sub-domains, and created inductive categories within the sub-domains. We used the mind-mapping software Freemind [31] to organize the data into a hierarchical structure of NASSS domains, sub-domains, inductive categories, condensed meaning units, and raw data.

We analyzed survey data using descriptive statistics (i.e., response frequencies for each question). We performed the analyses separately for the two pilot projects. We used the statistical software R [32] and the ggplot2 package [33] to perform the analyses and create diagrams.

The results from the qualitative analysis were triangulated with the results from the e-Ready surveys, and jointly used to classify each of the NASSS domains as simple, complicated, or complex, based on the definitions by Greenhalgh et al. [14]. In the final phase, we selected illustrative quotes to present and translated these to English.

## Results

### Patients’ and staff’s experiences of the HTH service

In the subsequent sections, we present the findings from the qualitative analysis of patients’ and healthcare staff’s experiences of the two pilot projects and relevant results from the e-Ready survey, organized around the seven NASSS domains (D1-D7). The responses to all questions of the e-Ready survey are presented in Additional file 1: Appendix 1. We have used the term “respondent” when referring to participants of the e-Ready survey, and the term “informant” when referring to interview or workshop participants. Where appropriate, informants are distinguished by role (i.e., patient, nurse, or staff).

#### D1. The complex nature of HF and COVID-19 conditions

We evaluated the conditions as complex. Informants described that the nature of COVID-19 was not yet fully understood, which caused uncertainty. Although staff were more familiar with HF, they described it as a condition with high variation in symptoms. In addition, comorbidities (e.g., diabetes) and socio-economic aspects posed challenges to care planning. Staff explained that new medications are often expensive and may not be affordable for all patients. Thus, they emphasized the importance of patients’ knowledge and financial stability as prerequisites for managing their health conditions.

Since not much is known about this illness [COVID-19], one doesn’t know so much about this illness, getting discharged can bring back this worry that one had before being hospitalized. And you hear all sorts of stories about someone going home and then once at home the disease comes back and all kinds of stuff. (COVID-19 patient 04)

If you have family, if you’re alone, and so on, for many it can steer their finances having to pick up the prescriptions that we write and assume they are taking. It is super important. Because not everyone has the money for it. (Staff, workshop 02)

#### D2. Technology facilitates communication and self-monitoring

We considered the technology to be complicated. Respondents were relatively inexperienced with working with digital technologies prior to the HTH pilot project (e-Ready question 1d). Although some had difficulties using the equipment (e.g., blood pressure cuff or thermometer), nurses and patients generally experienced the technology as user-friendly and easy to use after training. The most appreciated features were: the communication functionalities (i.e., chat, phone, and video) that made it easy to get in contact with the nurse; self-monitoring of health parameters; the history function, that enabled patients and nurses to get an overview of health parameter measurements and follow trends. Patients expressed that the information generated and made visible through these features provided them with a sense of safety and relief, as deviating measurements triggered alerts.

No, [the HTH equipment] was actually very simple to use. So, there were no difficulties. At first, I thought it would be difficult. Because I was thinking that I've never done anything like this before. But after using it a few times I thought, well, this is not that difficult, it's as easy as it can be. (Heart failure patient 03)

However, the informants also identified some limitations: the HTH service was only available in one language (i.e., Swedish), which reduced the number of potential users in a multi-national patient population; the equipment was large and clunky, which inhibited mobility; sometimes measurements were not reliable, which created uncertainty regarding dependability; and there was no integration with other systems (e.g., the electronic health record system or patients’ smartphone applications).

It was like having two separate systems. I worked in TakeCare [electronic health record system] and then it was a little… it had been easier if I could have gained direct access to this somehow. It would probably have made things a bit easier. (Nurse 01)

#### D3. The value proposition is simple for individuals

We interpreted the value proposition as simple from the perspective of staff and patients, and complicated from the organizational perspective, given a poor understanding of its potential cost-effectiveness. Our data did not capture the supply-side value (i.e., the value experienced by the supplier of the technology). Before the pilot project, the majority of respondents reported that they saw great or fairly great value in implementing HTH at their workplace (e-Ready question 9). More than half also believed that their ability to offer high quality care would be slightly better or much better (e-Ready question 5d). As the first pilot project started during the pandemic, staff saw a main value in the ability to provide remote care while reducing the number of physical visits at the hospital. This was later corroborated by patients with COVID-19, who valued the possibility to stay at home with an increased sense of safety.

Interviewer: What made you want to participate [in the pilot project]?

Patient: The deciding factor was this illness, COVID-19, which was such a new condition… so being able to keep this connection to the healthcare staff and feel that you’re just a keyboard’s tap away from getting help or contact – that is what made me feel like, alright, it’s okay, yes I can be released, I am going home but I still feel that if anything odd were to happen I’d have the possibility to quickly get in contact and get help (…) that feeling of safety; that is what I think is the key in all this. Safety that the patient, that I as a patient, feel even at home. (COVID-19 patient 04)

Patients with HF also experienced increased motivation to live a healthier life; one of them described how their daily self-monitoring had become a new routine, functioning as a complement to traditional care. However, there were also patients who were not sure about the value of the HTH service and participated in the pilot project to contribute to research and development. Nurses perceived that the HTH service aided in early detection of problems; they believed that continued use of the service could contribute to reduced inpatient care visits and patient suffering. The daily interactions with patients were time-consuming for the nurses, but as these interactions involved communication around health-related issues as well as friendly chatting, they felt that they got to know their patients quite well. They also believed that the knowledge generated through the HTH service was educational for patients, contributed to a shared understanding, and enabled patients and their carers to be more engaged in their care.

Interviewer: In what way do you think that HTH was helpful, apart from just monitoring weight?

Nurse: To nip it in the bud, that we could initiate a treatment early on instead of having [patients] feel unwell and you know, maybe not contacting us because they think they could wait one more day. (Nurse 01)

#### D4. Adoption requires new routines for patients and staff

We interpreted the adopter system as complicated because healthcare staff as well as patients had to implement new routines. Before the pilot project started, most respondents found there was quite extensive experience among colleagues in implementing new work routines (e-Ready question 1e). However, they did not expect the implementation of HTH to improve their ability to manage all their work; the majority of respondents believed this ability would remain unchanged or worsen (e-Ready question 5d). A nurse described how they only offered the HTH service to patients who they believed would be able to collaborate.

One challenge was the technical bit. Not on my part, but for the patients I mean (…) and it’s difficult to explain via telephone…. It meant that I had to go home to the patient and show them in-person and it took a bit of time since they live a bit far away from the hospital. So yeah, we didn’t always really have time for that, so we had to prioritize a bit differently. (Nurse 01)

Using the HTH service implied a shared responsibility for patient monitoring between patients and HCPs. Patients were expected to take self-measurements; HF patients once per day and COVID-19 patients several times per day. Patients felt that it was generally not difficult to integrate self-measurements in their daily routines but would have been a challenge with a full-time occupation. They described that they relied on the nurses to monitor their data and get in contact in case of deviations. Some perceived that the remote collaboration worked well, whereas others felt that it was difficult to get in touch with nurses as they only read chat messages once per day.

I had fixed times when I was supposed to do these checks. Eh, and also answer three questions…. So in the morning I took my saturation, I took my temperature, I took my blood pressure… and then I had a video call once a day.… It was said at 6 in the morning, 12 noon and 18 in the evening. At 6 o'clock in the morning didn’t really work because I did not get up to take [the measurements] at 6 but rather did it when I had woken up…. But 12 and 18 were not difficult to follow. (COVID-19 patient 05)

Nurses explained that apart from checking and responding to patients’ self-measurements and messages, they supported patients in solving technical problems, which required other skills than nurses had been trained in. They made home visits when patients needed support, and if needed, they could call the supplier’s helpdesk for additional technical support.

#### D5. Involving the organization requires a shared vision and sufficient resources

We classified the organization as complicated with elements of complexity. In terms of the organization’s capacity and readiness to innovate, respondents reported that the management clearly communicated the needs for the HTH implementation (e-Ready question 3a) and encouraged staff to participate in activities introducing HTH (e-Ready question 3b). However, in both pilot projects, more than half of the respondents believed that the manager was insufficiently or not at all aware of how HTH affects the current working routines (e-Ready question 3e). The majority reported that there was no or limited discussion among colleagues about how work routines would need to change (e-Ready question 4a), which duties that would need to be omitted (e-Ready question 4b), and new duties that would need to be performed (e-Ready question 4c); less than half believed that employees put much effort into working together to adapt current work routines (e-Ready question 4d) or taking a collective responsibility for the implementation of HTH (e-Ready question 4e). They perceived that the resources in terms of time and staff needed to introduce HTH were provided to a small extent or not at all (e-Ready question 1a-b). Staff felt that they were expected to prioritize the HTH pilot project on top of existing tasks or responsibilities, without being provided additional resources. Apart from preparing the equipment, which required a lot of effort, staff as well as patients had to be trained. A challenge for staff was that routines and processes could vary greatly between different parts of the hospital. They highlighted the need for collaboration and standardization, and a communication climate and leadership that would foster a shared vision among team members. Collaborations with other providers within and outside the hospital walls challenged the ability to assess the costs and benefits of a more large-scale implementation of HTH, which added an element of complexity.

Interviewer: What would be needed to get going a bit more with this, to not meet so much resistance?

Nurse: Above all, time. And it requires committed employees and management. Because we cannot have too many things to work against, one may have to focus on one thing… But it is very rare that we get to work like that; rather, we get a lot of inputs: ‘Now you have to do this, now you have to do that’. So we never have time to finish what we work on, because it takes time to change. (Nurse 02)

#### D6. Implementation in the wider system requires collaboration, not competition

We interpreted the wider system as complicated, although our data contained limited information regarding this dimension. There was no data that reflected the political, policy, and socio-cultural contexts. In terms of the regulatory context, staff experienced that, in general, laws and regulations could be barriers to the implementation of new technologies. In terms of the professional context, staff discussed that Sweden is lacking research about HTH, which makes it difficult to argue a case for implementing HTH more widely. They believed that more “hard numbers” are needed. Another challenge concerned interorganizational relations; as one of the patients said, it does not seem like their general practitioner and hospital staff have much contact. Informants emphasized that different healthcare levels and organizations need to collaborate in networked care, rather than compete against each other.

…for a while I felt that there was not so much collaboration [within the region] but more competition about who came first… Because you want that, if there is to be a broader context or something, you want to collaborate on this being an important function and how we work. The same patient can theoretically move between all care specialties (Staff, workshop 01).

#### D7. Embedding and adaptation is the future, but requires wide demand

We evaluated embedding and adaptation over time as complicated. Patients and nurses were positive about using and adapting HTH in the future, making it accessible to other patient groups and more flexible to use (e.g., by enabling platform agnostic implementation). Yet, staff also highlighted that there needs to be a larger demand from both patients and healthcare professionals to enable wider adoption and digital transformation of healthcare.

Interviewer: Would you recommend distance monitoring to other patients with COVID-19?

Patient: One hundred percent. Without doubt, for real, one has to start using this at large scale.

Interviewer: Why? What do you think that could lead to?

Patient: Well, this is how it is, I don’t occupy a bed at hospital. But still, I get all the care I need as if I were there. As I said in the beginning, have your cake and eat it, it’s like picking the best from both worlds so I recommend this one hundred percent and I am totally convinced that it will become commonplace… it will be just as if a doctor prescribes medication that one picks up at the pharmacy and uses at home… the equipment will follow along, or if we take it one step further, you can get everything as an app in your own smartphone. (COVID-19 patient 04)

Interviewer: And you mentioned that interest is important, do you mean from the patient’s side or from healthcare’s side?

Nurse: Both… Because patients need to meet engaged individuals, how else should they feel enthusiasm if they don’t have anyone that cheers them on and is active in this and explains the value and so forth… and then they need to take these measurements so we don’t need to call and ask them every day. But we didn’t have any problem with that. We got them, sometimes [the patients] overslept a bit, but it was no worry. (Nurse 02)

## Discussion

In this study the development and evaluation of an HTH service was based on QI and complexity frameworks. The development process led to two pilot projects of an HTH service for patients with HF and COVID-19. Patients and staff perceived the service as valuable as it enabled rapid feedback, and improved communication and collaboration between patients and HCPs. Yet, despite the extensive development efforts, there was a perceived gap between how individuals valued the service and the capacity of adopters, organization, and wider system to effectively integrate this service into routine care. The discussion below includes three main sections: first a review of the experience of using a theory-driven approach in the development of HTH service; a further analysis of interactions between the NASSS dimensions and how this influenced early adoption; methodological considerations.

### Theory-driven development of an HTH service

The theory-driven approach enabled to tailor the HTH processes to the needs of the specific patient groups, in the attempt to support the effective adoption of the HTH service.

The use of PDSA helped to adapt the HTH service after the inclusion of each new patient, and it was effective to learn how to develop the service stepwise to meet the needs of HCPs and patients. For instance, the need to use standard care plans was identified in one of the PDSA cycles (see section: Methods – The quality improvement process). Thus, this study supports the notion that PDSA, if used correctly [34], can inform the continuous development of clinical operations by reducing sources of artificial variation [28].

To our knowledge, this study was innovative in the attempt to combine PDSA and NASSS-CAT, which was done by conducting two NASSS-CAT workshops as two PDSA cycles. The use of the NASSS-CAT tools provided a complementary perspective as it helped to address the interactions between the domains more explicitly. While many of the challenges identified in the workshops were consistent with the lessons gained from PDSA cycles, the use of NASSS-CAT allowed for deeper insights into the perspectives of the multiple stakeholders involved. This allowed the creation of a shared understanding of the possibilities and challenges of using the HTH service in the hospital. Similarly, another Swedish study that applied NASSS-CAT found that its tools increased the awareness of complexity. The prospective application of NASSS-CAT allowed the identification of five project-specific recommendations to further develop the project before its deployment, and above all to identify the strengths and the challenges of future implementation. Two main issues in using the tool were also identified ex-post, i.e.: participants wished for a basic understanding of the assessment tool’s core concepts before using it; relevant stakeholders were excluded, such as the Information Technology department and staff members [18]. Participants in our study expressed similar challenges in relation to the use of NASSS-CAT.

Overall, the combined use of PDSA and NASSS-CAT was deemed helpful to support the learning needed to develop the HTH service in a hospital setting. However, even after two pilot projects, the service was not fully adapted to the needs of patients and staff, and the value for the organization was not emerging with clarity. The in-depth analysis of the data presented in this study allows for a deeper understanding of how the patterns of interactions between the NASSS domains may explain the struggles experienced to move from pilot projects to adoption, spread, and scale-up.

### Patterns of interactions between NASSS dimensions

The NASSS evaluation reported in this study elucidates on how the complex interplay between the context and the HTH service influenced its early adoption and future opportunities for spread and scale-up. Based on our assessment, most elements of the NASSS framework were simple (value for individuals), or complicated (technology, value for the organization, adopter system, organization, embedding and adaptation over time). The only dimension we deemed to be complex was the condition, and elements of complexity were identified in the organization domain. Thus, we found the struggles to move from pilot projects to adoption, spread, and scale-up, not to be due to the complexity of single elements, but rather to the inherent challenges of the interplay between some of them. Specifically, the interplay between the complexity of the condition and the technology, and the gap between the individual value and the organizational value.

#### Variation in value creation

HCPs and patients perceived that the service enabled early detection of deviations or problems for patients like Ethel, and improved communication and collaboration between patients and nurses. Thus, the self-monitoring of physiological parameters provided useful data for both patients and staff, in line with previous literature that identifies this as a key element of HF care [35]. Participants also valued the communication functionalities of the services, yet there is still limited knowledge of the effectiveness of phone calls and video communication for patients’ outcomes [35, 36]. The HTH service was valued both by patients who received it from outpatients’ units as well as by those who used it after discharge. While value was perceived by both groups, there was some variation. For COVID-19 patients the main value of using HTH was an increased sense of safety, whereas patients with HF could also experience that it could motivate them to live a healthier lifestyle. Yet, there is still limited knowledge on potential differences of using HTH to avoid hospital admission [37] or for early discharge cases [38].

The perceived value was also affected by some of the features of the technology in relation to the complexity of the condition. While the technology was overall deemed easy to use, various factors, including educational background and socio-economic factors may have influenced the eligibility to the study and the ability to use the technology as intended. Staff speculated that it was easier for COVID-19 patients to use the technology as they were generally younger patients. One aspect of the technology that hindered its use in the context of complex chronic conditions was the fact that it was perceived as large and clunky, thus affecting mobility. More research is needed to understand how HTH services can be effectively implemented to meet the needs of different patient groups, how patients can be effectively involved in the co-design of services [39, 40], and to assess the impact on quality of life [35, 41].

#### A gap between individual perceived value and organizational value

While both patients and HCPs could see the value of adopting HTH, there was a gap between the individuals’ perception of value and the organizational value. This gap could be explained by the overall low levels of readiness to adopt HTH, reflected in the perception that low resources were devoted to implementing the change and that managers were lacking awareness regarding its effects on current work routines. The organizational struggles were also reflected in the staff’s difficulty to integrate the new tasks into their daily work. Managers struggled to clearly communicate with staff about the needs of revising tasks and resources, and there was limited discussion on how HTH impacted work routines. To bridge the gap between individuals’ perceived value and organizational readiness, the findings suggest that more can be done to engage the staff in the discussion of how work processes and organizational capacity can be managed to integrate HTH-tasks into routine care. To support the internalization of the goal to adopt HTH, future implementation attempts could be enhanced by providing a platform to encourage frontline staff directly involved with the changes that are being implemented, to re-design the activities to fit the new routine and to co-design it as a group. Managers could use the perceived individual value to formulate a meaningful purpose, and then develop processes to facilitate the learning needed to address an evolving dynamic process where multiple challenges may arise with different levels of complexity. Recent studies of effective change in healthcare suggest that different levels of complexity must be addressed with different responses [42]. When complicated and complex situations arise, managers and facilitators should put efforts to work with staff to analyze the situation and plan plausible responses (with complicated challenges) and to engage and task staff with probing the situation, trying out different ideas, and studying the effects with a clear understanding that the problem may return (with complex challenges) [42–44].

The NASSS framework could also be enhanced by adding a component that reflects complexity of collaboration when tasks and responsibilities are shared. Indeed, the adoption of HTH services changed routines for both staff and patients. They shared responsibility for monitoring, which implied a mutual dependency. The shift from traditional care to HTH-supported self-management implicitly implies that tasks are shared to a higher extent, which also implies that the distinction between HCP and patient responsibilities gets blurred as these become more intertwined, similar to what has been reported in previous studies [45–47]. This interaction can contribute to complexity.

Finally, the gap between individual value and organizational value was widened by the inherent complexity of overcoming the fragmentation within the hospital and across providers, which is typical of patient care with complex conditions. The fragmentation of care processes complicates the assessment of cost-benefits that may be unevenly distributed across the units and organizations involved. Since the cost-benefits of using HTH for patients with chronic conditions is still not properly assessed, there is a need to view the adoption of these services as innovations, and to create the space for learning and conducting scientifically-sound evaluations. Such evaluations could help link the individual value perceived by staff and patients to the organizational and system value. The current COVID-19 pandemic has underlined the urgency to strengthen healthcare systems’ capability to implement eHealth services to continue providing care even when physical contact should be avoided. Thus, improvement efforts like the one studied here, need to be supported with institutional R&D funding to allow for explorations and learning and to gather real-life evidence of the organizational and system value of HTH services.

#### Organizational models to support the shift to HTH services

The challenge to integrate HTH tasks into clinical routine was complicated by several factors. In the HF pilot project, HCPs had to work on HTH in addition to their regular tasks, thus having to manage two parallel workflows. Another factor was that the planned care process, which should involve AHCaH home visits, did not materialize. The value of HTH is dependent on the ability to integrate it into current healthcare systems and routine care practices [6]. One way to achieve this is to integrate HTH into hospital care with mobile teams. Mobile teams aim at bridging the gap between hospital and primary care, specifically for frail elderly patients to develop long-term coordinated care plans with individually tailored interventions [48]. These plans can also include the use of remote-monitoring technologies and other elements of HTH. In this study, an approach similar to mobile teams was planned with AHCaH. However, as this was never implemented in the pilot project, the perceived fragmentation for the staff may have complicated the task integration even further. One plausible explanation for the failure to engage AHCaH was a perceived misalignment between the purpose of AHCaH and the patient needs. Successful implementation requires continual adaptation to align with the purpose of the organization and its users [12, 14, 17].

### Methodological considerations

The main strength of this study lies in the use of mixed methods to facilitate the development and evaluation of a HTH service. The research approach allowed for an in-depth understanding of the phenomenon. Yet, some limitations can be discussed.

The inclusion of two patient groups (i.e., COVID-19 and HF) was not originally planned, but happened as a consequence of the outbreak of COVID-19. The two target conditions differ, as COVID-19 was a new disease, for example with unknown medical risks, compared to HF. The study was not primarily designed as a comparison of the two pilot projects. Yet, in the analysis presented in this study, some differences were observed and thus captured in the writing.

Conducting research on one’s own organization has an inherent risk of bias. We attempted to mitigate this through triangulation of multiple data sources and by involving researchers external to the pilot projects in the data collection and analysis. Moreover, we carried out cross-referencing and validation with department managers, journaling, and continual reflection among co-authors without connections to the department. Moreover, the adherence to a solid and established framework such as NASSS throughout the data collection and analysis processes supported the comprehensiveness and completeness of the data and guided the analysis to include the relevant aspects and stakeholders as much as possible.

Regarding the interviews, they were semi-structured and based on interview guides adapted from the NASSS-CAT INTERVIEW tool [15]. Thus, they followed a theoretical framework that allowed for completeness and comparability. In addition, the qualitative content analysis was performed by three of the authors and then reviewed by two of the others, in order to reduce any potential personal bias or subjectivity in the coding.

Regarding the survey data, a limitation comes from the relatively low response rate and consequent lack of representativeness. To account for this, the quantitative analysis was only descriptive, and no inference testing was performed. Thus, survey data supported the understanding of the features and attitudes of a part of the staff of SH, but were not used to make general claims on the HCPs population.

The use of NASSS-CAT tools required extensive work to be translate into Swedish, to set up workshops, and to analyze the data. Indeed, it was only in the evaluation phase that data from the workshops and NASSS-guided interviews were thoroughly analyzed.

## Conclusion

The development and adoption of HTH services in hospital settings is a complex process. The combined use of PDSA, NASSS and NASSS-CAT can be helpful to develop and evaluate a HTH service, so that it is perceived as valuable by individual patients and staff. PDSA supports the systematic identification and resolution of unnecessary variation; NASSS and NASSS-CAT provide insights into how to manage the interaction among technology, adopters, organization, and the wider system. For the successful adoption of HTH services into clinical practice, the conditions must be created for learning, involving all stakeholders. The value for individuals must be supported by organizational efforts to learn how to integrate new routines and tasks into clinical practice and daily life, and how to coordinate multiple providers within and outside the hospital walls. Facilitating the learning processes that address this complexity can help increase the perceived readiness to adopt HTH and narrow the gap between the value for individuals and the value generated for the organization.

## Supplementary Information

**Additional file 1: Appendix 1.** E-Ready survey results.

## Data Availability

The datasets used and/or analyzed during the current study are available from the corresponding author on reasonable request.

## Abbreviations

AHCaH: Advanced Hospital Care at Home
CAT: Complexity assessment tool
CU: Cardiology unit
HF: Heart failure
HTH: Home telehealth
HCP: Healthcare professionals
IIM: Infections and Internal medicine (unit)
MMU: Multimorbidity unit
NASSS: Non-adoption, Abandonment, Scale-up, Spread and Sustainability (framework)
PDSA: Plan-Do-Study-Act (cycle)
QI: Quality Improvement
SH: Södertälje Hospital
