# Supplementary figures and images for: Mind the gap: analysis of two pilot projects of a home telehealth service for persons with complex conditions in a Swedish hospital
Source: BMC Health Serv Res. 2023 May 9;23:463. doi: 10.1186/s12913-023-09409-4 (PMC10169294; doi:10.1186/s12913-023-09409-4)

# Appendix 1

E-Ready survey results


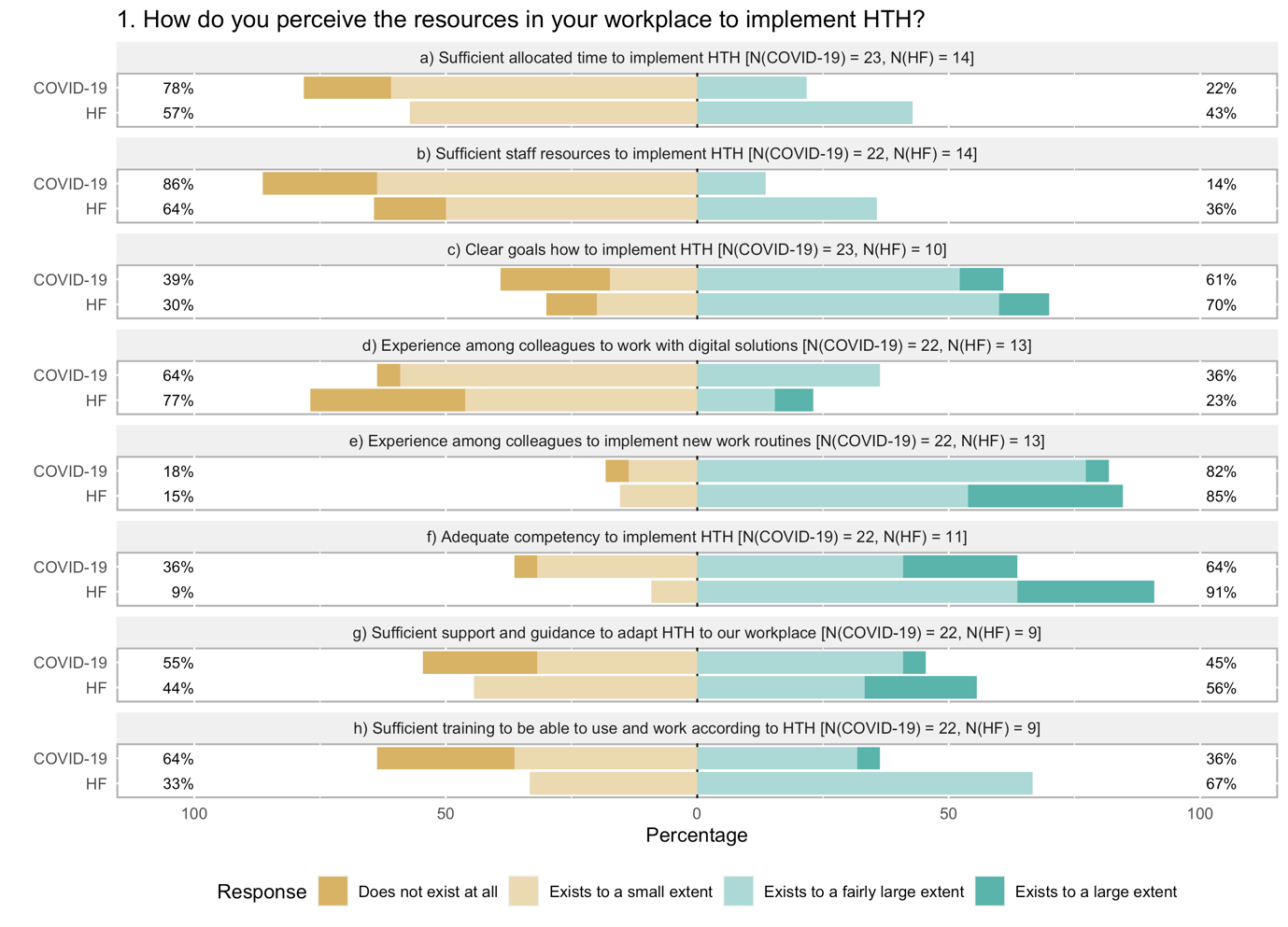

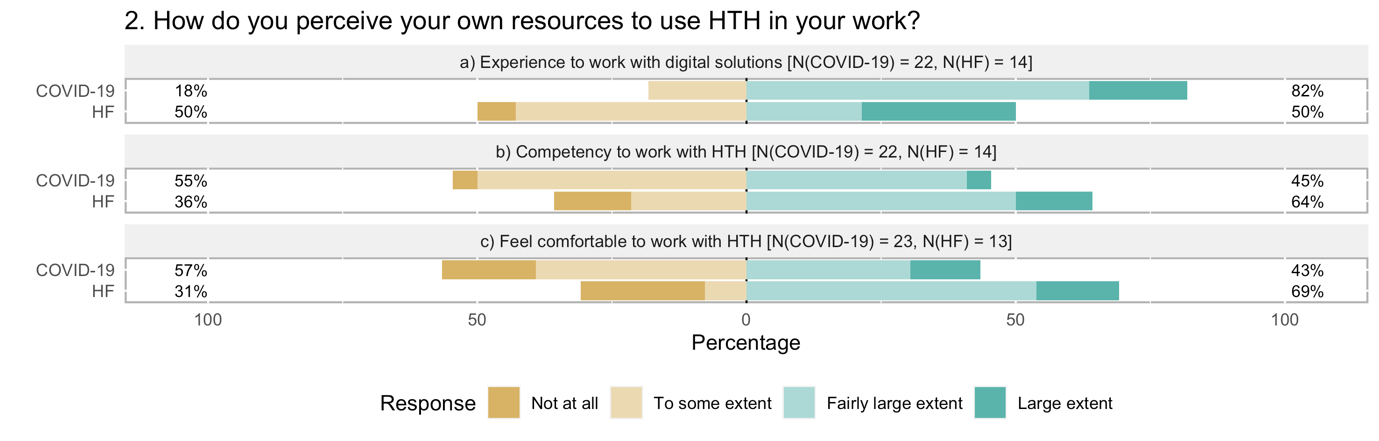

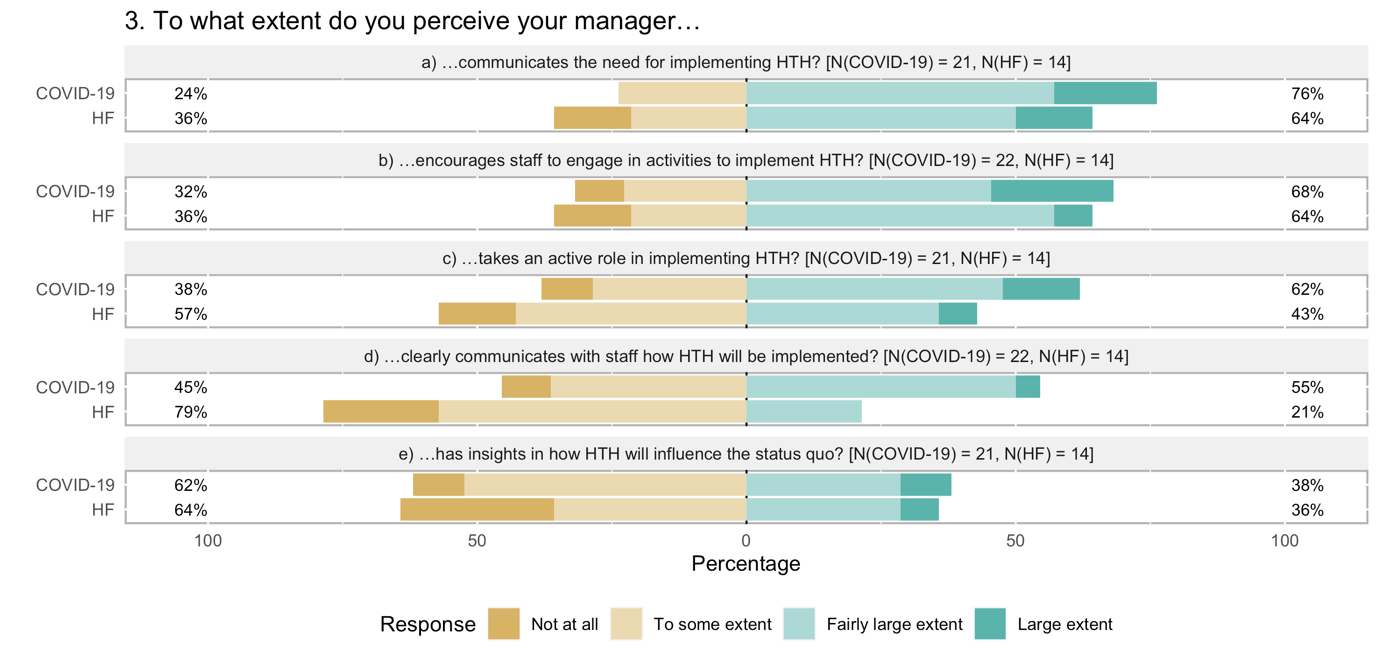

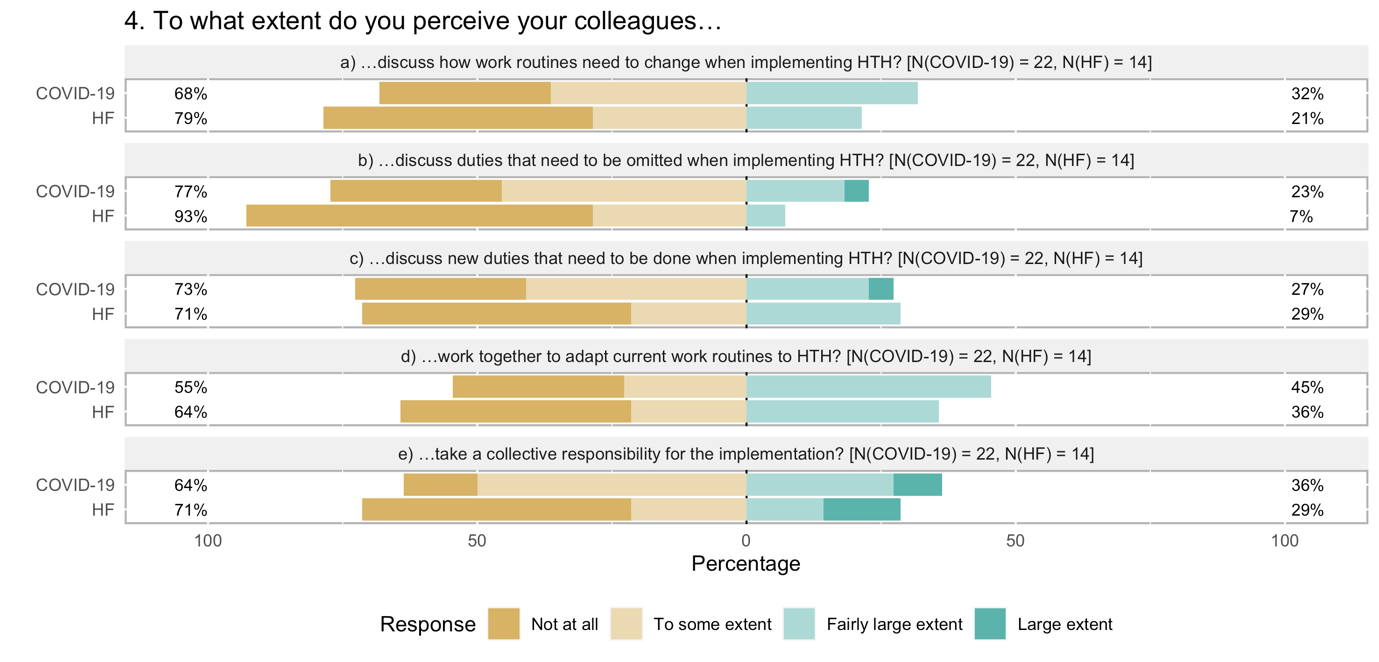

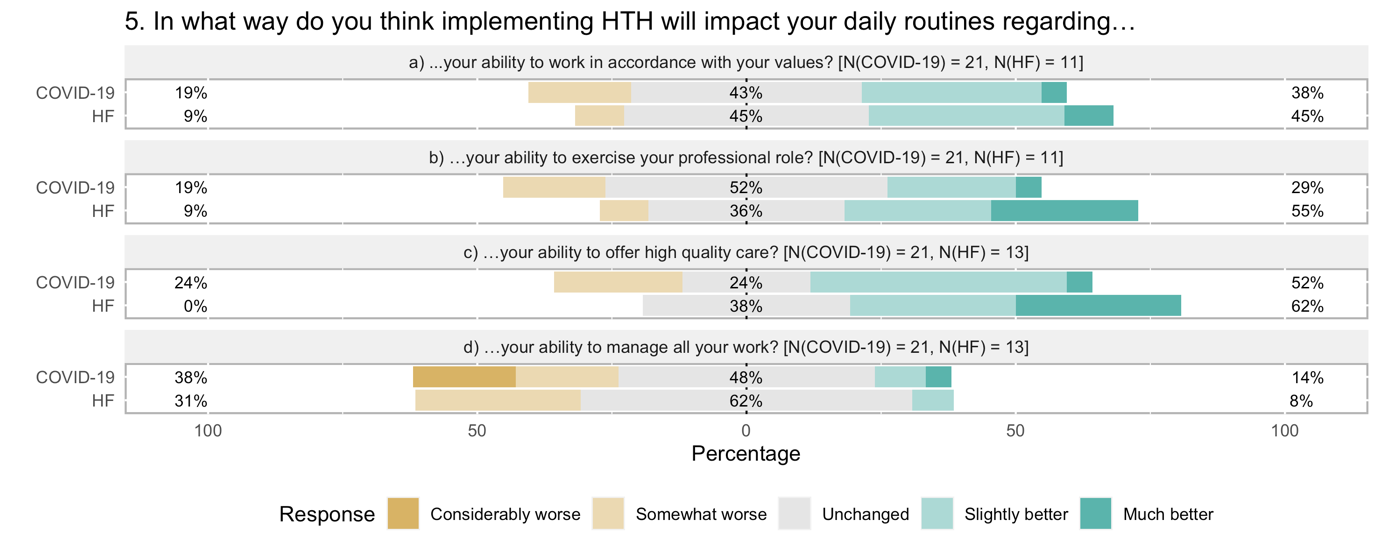

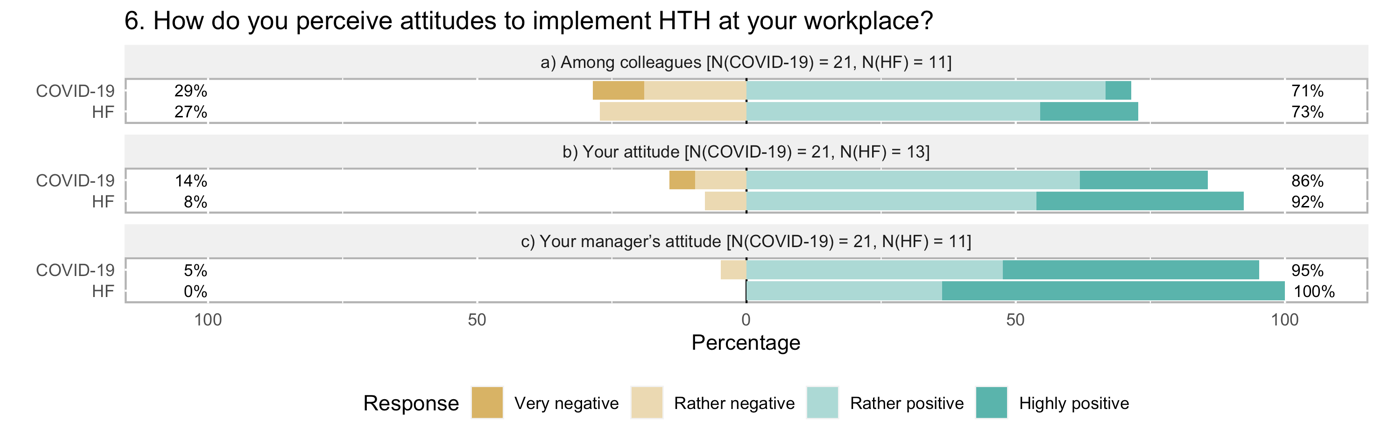

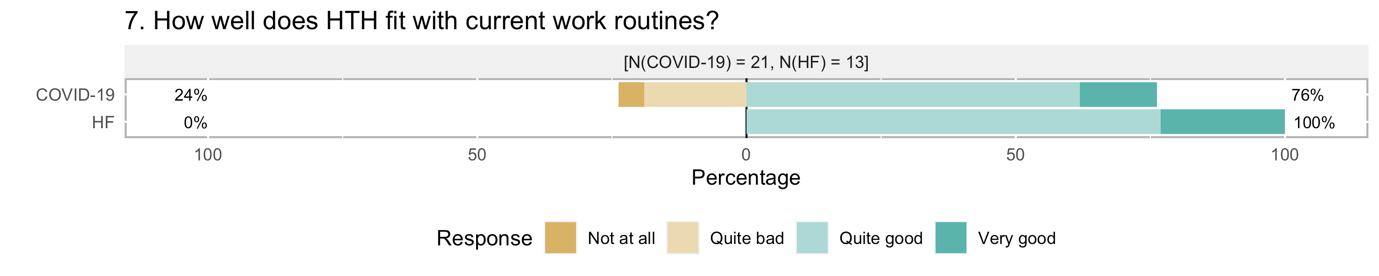

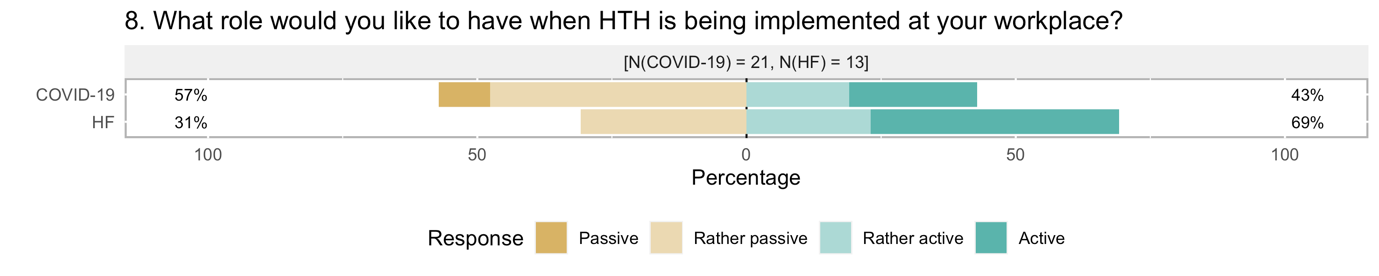

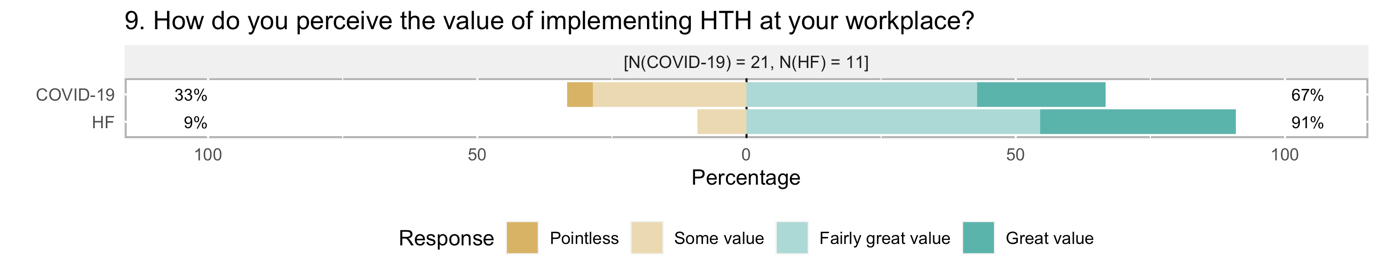

Supplement: Supplementary file 1 — Additional file 1: Appendix 1. E-Ready survey results. [file 12913_2023_9409_MOESM1_ESM.docx]
